# A Rare Case of Acute Methotrexate Toxicity Leading to Bone Marrow Suppression

**DOI:** 10.1155/2024/7693602

**Published:** 2024-03-15

**Authors:** Samreen Khuwaja, Matthew Lyons, Beenish Zulfiqar

**Affiliations:** ^1^Sam Houston State University, Academic Hospitalist Christus St Elizabeth Hospital, 2830 Calder Ave, Beaumont, Texas 77702, USA; ^2^Department of Medicine, Division of Connective Tissue Diseases, The University of Tennessee Health Science Center, 920 Madison Ave, Memphis, TN 38163, USA; ^3^Rheumatology VA Medical Center, 1030 Jefferson Ave, Memphis, Tennessee 38104, USA

## Abstract

Methotrexate is a first-line disease modifying antirheumatic drug used for the treatment of inflammatory arthritis. Bone marrow suppression is a common adverse reaction of methotrexate following its long-term use. However, low dose methotrexate is rarely associated with life-threatening bone marrow suppression. This case represents an atypical presentation of acute bone marrow suppression shortly after initiating treatment with low-dose methotrexate. A 76-year-old male patient presented with oral ulcers, poor oral intake, and acute kidney injury within 3 weeks of initiating 15 mg weekly of methotrexate for seronegative rheumatoid arthritis. Complete blood count was suggestive of pancytopenia with hemoglobin of 10.8 g/dL, total white cell count 3.36 (1000/uL) (absolute neutrophil count 490 micro/L), platelets 19,000, serum albumin 3.1 g/dL, ESR elevated at 83 mm/hr, CRP elevated at 86.6 mg/L, and ferritin mildly elevated at 625 ng/mL. Peripheral blood smear showed signs of bone marrow suppression but no signs of hemolysis or inflammation. Serum methotrexate levels were minimally detectable at 0.05 umol/L. Methotrexate was held, within 48 hours of admission; his WBC dropped to 1.48, Hgb 9.9, and platelets 15,000. ANC reached a nadir of 220. He was treated with broad spectrum antibiotics, high-dose folic acid, fluconazole for oral thrush, and intravenous bicarbonate and leucovorin supplementation, dosed at PO 20 mg daily. On day 7, his blood count showed improvement along with improvement in his symptoms. The patient was discharged home on day 8^th^ of hospitalization and upon one month follow-up in rheumatology clinic, his complete blood count had normalized. This case highlights multiple risk factors that triggered pancytopenia in our elderly patient, resulting in acute methotrexate toxicity.

## 1. Background

Methotrexate (MTX) is widely used in the treatment of inflammatory rheumatic disorders. However, adverse events and toxicities may occur with a wide range of severity indices, which can be dose or regimen dependent. Methotrexate is being commonly used in weekly dosage form for the treatment of rheumatoid and seronegative inflammatory arthritis. Low-dose methotrexate can rarely cause bone marrow suppression. The most common predisposing factors associated with bone marrow toxicity are the concomitant use of NSAIDs (nonsteroidal anti-inflammatory drugs), liver disease, immunosuppressants, underlying renal function impairment, low albumin, dehydration, etc.

This is the case of a 76-year-old elderly male, initiated on methotrexate 15 mg weekly for seronegative RA, who then presented with oral ulcers, poor oral intake associated with odynophagia, acute kidney injury, and pancytopenia possibly secondary to acute toxicity from methotrexate.

## 2. Case Presentation

A 76-year-old elderly male presented to the emergency department with complains of difficulty eating, puffiness around his eyes, and tender maxillofacial swelling. The patient was recently established at our Rheumatology Clinic for the treatment of potential seronegative rheumatoid arthritis, now progressing to worsening inflammation of small and medium joints. He was started on methotrexate 15 mg weekly with 1 mg of folic acid daily, approximately 3 weeks prior to presentation, after his promising response to the prednisone trial. His medication list comprised 15 mg of methotrexate to be taken by mouth weekly, 1 mg of folic acid daily, and allopurinol 200 mg. The patient also had a history of gout and osteoarthritis for approximately 10 years.

On examination, he was alert, awake, and oriented. Vitals were noted at a low-grade temperature of 100.8 F; otherwise, hemodynamically stable. Oral cavity had mucositis, with ulceration at the roof of the mouth. He also had developed oral thrush. He did not have any joint pain or swelling.

## 3. Investigations

His blood investigations showed pancytopenia with hemoglobin of 10.8 g/dL, total white cell count 3.36 (1000/uL) (absolute neutrophil count 490 micro/L), and platelets 19,000. He had no cytopenia noted on the laboratory investigations performed at his initial clinical visit. His liver function test showed total bilirubin 1.1 mg/dL, ALT 12 U/L, AST 20 U/L, ALP 171 U/L, and serum albumin 3.1 g/dL. Urinalysis showed only mild proteinuria of 30 mg/dL, and viral markers (HBsAG, anti-HCV, HIV, influenza A/B, RSV, and COVID-19) were all negative. Other investigations showed PT/INR 1.14, ESR elevated at 83 mm/hr, CRP elevated at 86.6 mg/L, and ferritin mildly elevated at 625 ng/mL. Peripheral blood smear showed signs of bone marrow suppression but no signs of hemolysis or inflammation. CT face/neck showed left frontal sinusitis and bilateral mastoid air cells/mastoiditis. Serum methotrexate levels were minimally detectable at 0.05 umol/L. Within 48 hours of admission, his WBC dropped to 1.48, Hgb 9.9, and platelets 15,000. ANC reached a nadir of 220 (Table 1).

## 4. Treatment

Patient's presentation was most consistent with methotrexate toxicity with immunosuppression and neutropenic fever along with acute kidney injury. His treatment was initiated with broad spectrum antibiotics (vancomycin and cefepime), high-dose folic acid 5 mg daily, fluconazole for oral thrush, and intravenous bicarbonate was initiated for urine alkalinization to enhance renal excretion of methotrexate and to prevent its precipitation in renal tubules to avoid nephrotoxicity. On day 6 of admission, he was treated with leucovorin supplementation, dosed at PO 20 mg daily.

## 5. Outcome and Follow-Up

Over the following days, he reported some improvement in oropharyngeal pain with improved swallowing as well, with symptomatic management, fluconazole, and antifungal mouthwash. His acute kidney injury resolved with IVF and improved oral intake.

His ANC improved over the following days from a low of 220 to 740 on Day 8. WBC had improved to 2.68 1000/uL, Hg stable at 9.5 g/dL, and platelets had normalized to 170,000. By that time, he was clinically improved with adequate PO intake, resolved fever curve, and no signs of infection. He was deemed stable for discharge and was sent home with plans to follow closely for repeat lab work in 7 days and Rheumatology Clinic follow-up within 1-2 weeks to further consider alternative medication options for inflammatory arthritis following intolerance to methotrexate. He was also instructed to hold allopurinol and any other potentially myelosuppressive medications while continuing daily folic acid supplementation. One month after discharge, ANC improved to 4510. WBC had improved to 7.21 1000/uL, Hg improved to 10.3 g/dL, platelets had normalized to 254,000.

## 6. Discussion

Methotrexate is a very commonly used DMARD (disease-modifying antirheumatic drug). It has both immunosuppressive and immunomodulatory effects. It acts as a folate antagonist via its antimetabolite mechanism and inhibits DNA synthesis [1]. MTX was traditionally developed to inhibit de novo synthesis of purines and pyrimidines in order to halt DNA and RNA synthesis in malignant cells. However, this mechanism may also have a contribution in its anti-inflammatory action, which makes its use the gold standard in RA therapy. MTX in its polyglutamate form can inhibit aminoimidazole-4-carboxamide ribonucleotide (AICAR) transformalyse (ATIC), which causes intracellular accumulation of AICAR. AICAR inhibits adenosine deaminase and adenosine monophosphate (AMP) deaminase. This in turn causes the release of adenosine nucleotides into the extracellular space. These nucleotides are converted into adenosine via a complex series of enzymatic reactions. Adenosine is a strong stimulus for a family of adenosine receptors (*A*_1*a*_, *A*_2*a*_, *A*_2*b*_, and *A*_3_), which has inhibitory effects on almost all inflammatory cell types [2].

MTX is widely used in the treatment of autoimmune rheumatic disorders such as rheumatoid arthritis, psoriasis, and various forms of lymphomas [3]. Most common side effects of methotrexate therapy are nausea, vomiting, and diarrhea, which can be reduced by folic acid supplementation. However, MTX toxicity may be influenced by higher body mass index (BMI), older age, high MCV, reduced creatinine clearance, prior history of GI events secondary to MTX use, low albumin, and female sex [4, 5]. MTX is widely excreted from the kidneys and its excretion rate is almost 85% within 48 hours of ingestion. Higher creatinine levels and dehydration can affect renal excretion of MTX. NSAIDs, proton pump inhibitors, and trimethoprim-sulfamethoxazole, gemfibrozil, probenecid, and penicillin derivatives may also lead to myelosuppression by reducing clearance or by displacing MTX from the protein binding sites [6].

The incidence of MTX-induced pancytopenia is low, i.e., 1.4% [7]. In a retrospective study of 70 patients presented, data on MTX toxicity elucidated serious complications secondary to pancytopenia in all cases reported between 1980 through 1995. The mean cumulative dose of MTX in these 70 patients was 675 mg. Upon reviewing their home medication list, 40 out of 70 patients received slow-acting antirheumatoid drugs (SAARDs) prior to MTX therapy, and 58 patients received other drugs such as NSAIDs, antibiotics, diuretics, antacids, antidepressants, digoxins, antihypertensives, and antiepileptic medications. At least 12 patients were on more than 5 drugs at the same time. 58% of the patients (38 cases) had underlying renal abnormalities and 51% of the cases (36 cases) had some type of major or minor infection with sepsis secondary to E.coli, *Staphylococcus aureus*, and Hemophilus influenza. Low album levels were noted in 24% (17 cases) and abnormal LFTs in 10% (7 cases). Only 6% (4 cases) were diabetics and less than 5% were obese and had a history of amyloidosis or alcoholism. Pancytopenia was fatal in 12 patients with a mean age of 66.8 and female predominance. The minimum cumulative dose was 10 mg in only 2 patients who did not survive. This study largely focused on the factors leading to MTX toxicity, which identified that most patients had impaired renal function, polypharmacy, underlying infections, and low albumin levels [8].

BM toxicity secondary to MTX can be an idiosyncratic, dose-dependent cumulative effect if the drug has been taken for years or can be related to a genetic polymorphism of A1298C and C677T MTHFR (methyl tetra hydro folate reductase) gene [8–11]. Polymorphisms of these genes can develop a wild type variant which may decrease the activity of MTHFR and thus increase the risk of toxicity [12]. Various studies have been conducted to elaborate the role of A1298c (AA and CC types) and C677T (TT and CC type) gene polymorphism of MTHFR to determine a potential etiology of MTX related toxicity in RA patients. In a cross-sectional study, conducted on 93 RA patients, the allele and genotype distribution of A1298cc and C677T was compared with 377 healthy patients. Variables noted were age, gender, dose of MTX, treatment with folic acid, C-reactive protein, number of tender and swollen joints, and hours of morning stiffness. The study found a protective effect of A1298CC with a lower rate of MTX-induced side effects. However, there was no significant association found between C677T- and MTX-related adverse events [13]. In contradiction to this, at least three separate studies conducted on patients diagnosed with chronic myelogenous leukemia, breast cancer, and ovarian cancer concluded that the C677TT type is associated with severe MTX-induced hyperhomocysteinemia and may induce toxicity [12, 14, 15]. Hence, homocysteine levels may serve as a marker to detect MTX-induced toxicity in susceptible individuals.

Our case highlights multiple risk factors that triggered pancytopenia in our elderly patient. Patient's age placed him at risk for BM toxicity, which may have been influenced by his low GFR. Albumin is low in patients with RA, likely secondary to its depletion and consumption in the setting of inflammation and poor nutritional status. As 42–57% of MTX is bound to albumin, hypoalbuminemia leads to increased levels of free MTX, causing an increased risk of BM toxicity [6]. Patient's albumin levels were lower than normal, ranging between 2.7 and 3.1 g/dL. Patient's first presenting symptom was mucositis, which may have led to decreased oral intake, causing prerenal acute kidney injury. Reduced renal function is the most important factor causing MTX toxicity. Patient was on allopurinol, which may have contributed to myelosuppression as well in the setting of his acute illness. As per guidelines, our patient was appropriately treated with MTX 15 mg weekly after a brief course of steroids and was followed weekly as per guidelines by ACR (American College of Rheumatology), as short-term glucocorticoids are often necessary to alleviate the symptoms before initiating DMARDs in DMARD-naïve patients with moderate-severe disease activity [15]. Despite of his normal renal function, MCV, and not so critically low albumin, he still developed significant side effects after only 2 doses of MTX, which suggests an idiosyncratic mechanism, possibly related to a predisposing genetic factor. Further head-to-head clinical trials are necessary to identify patients at risk for idiosyncratic reactions and genetic polymorphisms leading to life-threatening MTX toxicity.

## 7. Conclusion

We believe that the abovementioned case represents an unusual presentation of acute methotrexate toxicity in an elderly patient. While rare, it is important to recognize various factors leading to such adverse reaction. Further head-to-head clinical trials are necessary to identify patients at risk for idiosyncratic reactions and genetic polymorphisms leading to life-threatening MTX toxicity.

## Figures and Tables

**Table 1:** Laboratory Data.

Days	Day 1	Day 2	Day 3	Day 4	Day 5	Day 6	Day 7	Day 21
WBC	3.70	3.36	1.65	1.48	1.24	1.50	2.68	7.21
RBC	3.74	3.48	3.35	3.26	3.19	3.03	3.10	3.41
Hemoglobin	11.4	10.8	10.4	9.9	9.1	9.1	9.5	10.3
Platelets	22	19	15	16	24	34	170	254
Neutrophil %	69.2	58.4	Not performed	Not performed	17.7	Not performed	27.7	62.5
Neutrophil #	2.41	1.96	Not performed	Not performed	0.22	Not performed	0.74	4.51
Creatinine	1.77	1.28	1.08	0.99	1.06	1.00	1.13	1.04
Albumin	3.3	3.1	Not performed	Not performed	2.7	Not performed	Not performed	3.4

Normal ranges: WBC: 4.5–11.0 × 10^9^/L, RBC: 4.7–6.1 cells/mcL, hemoglobin: 13.8–17.2 g/L, platelets: 150,000–450,000/micro/L, neutrophil %: 40–60%, absolute neutrophil count: 2500–6000, creatinine: 0.7–1.3 mg/dL, albumin: 3.4–5.4 g/dL.
